# Risk Factors for Post-operative Planned Reintubation in Patients After General Anesthesia: A Systematic Review and Meta-Analysis

**DOI:** 10.3389/fmed.2022.839070

**Published:** 2022-03-09

**Authors:** Zhiqin Xie, Jiawen Liu, Zhen Yang, Liping Tang, Shuilian Wang, Yunyu Du, Lina Yang

**Affiliations:** ^1^Department of Nursing, First Affiliated Hospital of Nanchang University, Nanchang, China; ^2^School of Nursing, Nanchang University, Nanchang, China

**Keywords:** general anesthesia, risk factor, meta-analysis, reintubation, systematic (literature) review

## Abstract

**Background:**

The occurrence of postoperative reintubation (POR) in patients after general anesthesia (GA) is often synonymous with a poor prognosis in patients. This is the first review analyzing scientific literature to identify risk factors of POR after general anesthesia. The purpose of this study was to collect currently published studies to determine the most common and consistent risk factors associated with POR after GA.

**Methods:**

We have retrieved all relevant research published before April 2021 from PubMed, Embase, Web of Science, and the Cochrane Library electronic databases. These studies were selected according to the inclusion and exclusion criteria. The Z test determined the combined odds ratio (OR) of risk factors. We used OR and its corresponding 95% confidence interval (CI) to identify significant differences in risk factors. The quality of the study was evaluated with the NOS scale, and meta-analysis was carried out with Cochrane Collaboration's Revman 5.0 software.

**Results:**

A total of 10 studies were included, with a total of 7,789 recipients of POR. We identified 7 risk factors related to POR after GA: ASA ≥ 3 (OR = 3.58), COPD (OR = 2.09), thoracic surgery (OR = 17.09), airway surgery (OR = 9.93), head-and-neck surgery (OR = 3.49), sepsis (OR = 3.50), DVT (OR = 4.94).

**Conclusion:**

Our meta-analysis showed that ASA ≥ 3, COPD, thoracic surgery, airway surgery, head-and-neck surgery, sepsis and DVT were associated with POR after GA.

**Systematic Review Registration:**

https://www.crd.york.ac.uk/prospero/display_record.php?, Identifier: CRD42021252466.

## Introduction

Postoperative reintubation (POR) refers to intubation after extubation failure following general anesthesia (1). Indications for POR range from acute airway compromise to postoperative cardiac, respiratory or mental status complications (2). Given the wide range of clinical scenarios, the timing of POR after surgery varies from occurring immediately after extubation in the operating room to after several postoperative days (3). POR is still a significant adverse event after general anesthesia using endotracheal intubation since it has negative consequences for the patient, including longer intensive care unit (ICU) length of stay (LOS), higher related morbidity and mortality (4). Therefore, it is imperative to prevent the occurrence of POR in patients following GA.

The causes of POR can be divided into respiratory causes and non-respiratory causes. The former include hypoxia, respiratory muscle weakness, muscle relaxant residue, airway obstruction, phrenic nerve injury, to name a few, while the latter include accidental catheter prolapse, hemodynamic imbalance, unexpected change of operation, to name a few (5–7). Many previous studies have explored POR risk factors in patients undergoing different types of surgery under general anesthesia. However, due to limited sample size and demographics, the study results remain highly controversial.

To that end, we are first to review and perform a meta-analysis of the risk factors associated with POR in patients after GA.

## Materials and Methods

This meta-analysis was registered on Prospero with the registration number of CRD42021252466 and performed according to the PRISMA statement and the Meta-analysis of Observational Studies in Epidemiology (MOOSE) guidelines (8, 9).

### Search Strategy

We conducted a meta-analysis of all English language articles using the Cochrane Library, PubMed, Embase and Web of Science databases. The search included all relevant reports before January 2022, the date of the initial search is January 8, 2022. Additional records were identified by contacting authors and searching reference lists from the literature. This study followed the PICO statement (Table 1), and the search terms included “reintubation,” “re-intubation,” “factors,” “risk factor,” “influence factor,” “relevant factor,” “Anesthesia,” “surgery,” and “operative”. We showed the search strategy in PubMed database is available in Table 2. The full search strategies for all databases are available in Supplementary Material 1. We screened records from the titles and abstracts to full-text articles, discarding unrelated publications and duplicate records.

**Table 1:** The PICO statement about study.

**PICOS**	**Abbreviation**	**Elements**
Patient population	P	Patients after surgery under general anesthesia
Intervention/exposure	I	Postoperative reintubation
Comparison/control	C	Postoperative non-reintubation
Outcome	O	Risk factors of postoperative reintubation
Study design	S	Cohort study,case control study, cross-sectional study

**Table 2:** Search strategy in PubMed database.

**Number**	**Search terms**	**Results**
#1	(reintubation[Title/Abstract]) OR (re-intubation[Title/Abstract])	2735
#2	(((factors[Title/Abstract]) OR (risk factor[Title/Abstract])) OR (influence factor[Title/Abstract])) OR (relevant factor[Title/Abstract])	2470943
#3	((Anesthesia[Title/Abstract]) OR (surgery[Title/Abstract])) OR (operative[Title/Abstract])	1646586
#4	#1 and #2 and #3	367

### Inclusion and Exclusion Criteria

The inclusion criteria were as follows: (1) Types of studies: observational studies including cohort, case-control and cross-sectional studies; (2) Types of participants: patients who have undergone POR after GA; (3) Types of comparison: comparisons of risk factors for POR after GA.

The following types of records were excluded: (1) records with incomplete data; (2) nonoriginal studies (conference abstracts, editorials, letters, reviews, meta-analysis, commentaries or case reports); (3) records rated 4 and below by the NOS. Two reviewers independently assessed the eligibility of all studies. A consensus was reached through discussion in cases of disagreements.

### Data Extraction and Quality Appraisal

We used standardized data collection tables to extract the following information: author name, year of publication, country, study design, study period, number of patients in cases and controls, risk factors, odds ratio (OR), and study quality.

The quality of the studies was evaluated according to the Newcastle-Ottawa Scale (NOS) (10). NOS consists of 8 items, divided into three dimensions: selection, comparability and outcome. The NOS scale ranges from 0 to 9. Studies were considered to be of high quality if they obtained a score of seven or more.

Two reviewers (LJW and TLP) independently conducted the data extraction and study quality assessment. Disagreements were resolved through discussion.

### Statistical Analysis

Review Manager 5.3 (the Nordic Cochrane Center, Copenhagen, Denmark) software was used for statistical analysis of the data. We would conduct a meta-analysis if a risk factor was reported by at least two studies using multivariate regression analysis. The pooled odds ratio (OR) was calculated to evaluate the risk factors of bivariate variables of POR after GA. *Z* test was used to determine the significance of the difference. χ^2^ test and *I*^2^ statistics were used to evaluate the heterogeneity in the analysis. A value of *I*^2^ of 0–25% represents insignificant heterogeneity, 26–50% low heterogeneity, 51–75% moderate heterogeneity and 76–100% high heterogeneity (11). The random-effect model was used to calculate the 95% confidence interval of the combined effect and its homogeneous data (*I*^2^ > 50% or *P* < 0.05). Otherwise, the fixed-effect model was adopted. A sensitivity analysis was carried out by excluding individual study sequentially and compare the pooled results by using a fixed effect model and a random effect model. When there is no significant difference in the *P*-value of the corresponding combination effect, the result can be considered robust. Harbord's test of Stata Version 15.0 (StataCorp, College Station, TX, USA) was used to check publication bias. *P* > 0.1 indicates no publication bias in this study (12). The combined exposure rate of the control was used to replace the overall population exposure rate. To calculate the The population-attributable risk proportion (PARP), we used the following calculation formula: PARP = Pe (OR-1)/ [Pe (OR-1) +1].

## Results

### Study Selection

**Figure 1:**
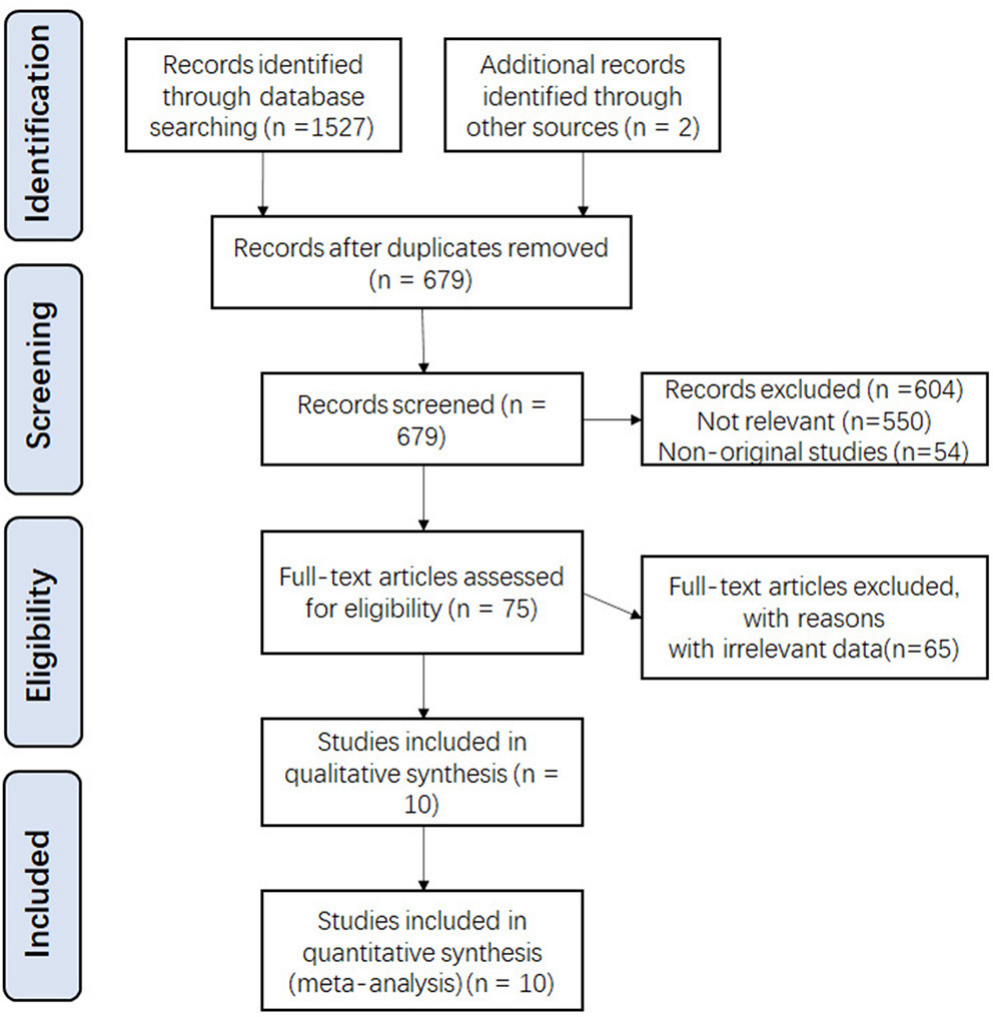
Flow chart of the selection process.

According to our search strategy and other sources, a total of 1,529 potentially relevant studies were identified. Among them, 850 were excluded due to duplication, 604 were excluded after screening the title and abstract, and 65 were excluded after full-text review because they did not meet the criteria. Finally, a total of 10 studies and 5,15,940 patients were included in this meta-analysis. (1, 3, 13–19) The selection process and results are shown in Figure 1.

### Characteristics of the Studies

The included studies were published between 2010 and 2021. Five studies were conducted in the United States, four in China and one in Thailand. The sample sizes included in the study ranged from 180 to 3,16,640. All identified studies followed an observational design: nine were case-control studies, while one was a retrospective cohort study. Table 3 summarizes the characteristics and quality evaluation of the included study following the NOS quality evaluation scale.

**Table 3:** Characteristics of studies included in the meta-analysis.

**References**	**Year**	**Type**	**Country**	**Study period**	**Cases/** **controls**	**Quality assessment**	**Risk factors**
Chen et al. (1)	2021	Case-control	China	2014–2018	36/144	7	Male gender, age>65, thoracic surgery, asa≥3, head-and-neck surgery
Brovman et al. (3)	2017	Cohort	USA	2007–2014	6936/309704	6	Age>65, COPD, ASA≥3, sepsis, male gender, emergency case, DVT
Lin et al. (17)	2013	Case-control	China	2005–2009	130/390	7	COPD, ASA≥3, airway surgery, head-and-neck surgery, rocuronium
Greco et al. (15)	2016	Case-control	USA	2008–2015	39/222	8	Male gender, rocuronium, emergency case
Wilson et al. (19)	2020	Case-control	USA	2002–2011	100/47325	7	Male gender, ASA≥3, COPD
Jian et al. (16)	2013	Case-control	China	2004–2012	97/1147	7	COPD
Ting et al. (20)	2010	Case-control	China	2005–2007	83/249	6	COPD, head-and-neck surgery, airway surgery
Ramos et al. (13)	2017	Case-control	USA	2005–2014	182/9552	8	Sepsis, DVT
Rafael De la Garza et al. (14)	2017	Case-control	USA	2007–2013	22/1228	7	NA
Rujirojindakul et al. (18)	2012	Case-control	Thailand	2001–2011	164/656	8	Age>65, thoracic surgery, ASA≥3, airway surgery, head-and-neck surgery, emergency case

*NA, not available*.

*Low-quality research, 0–4 points; medium-quality, 5–6 points; high-quality research, 7–9 points*.

### Risk Factors of POR

**Figure 2:**
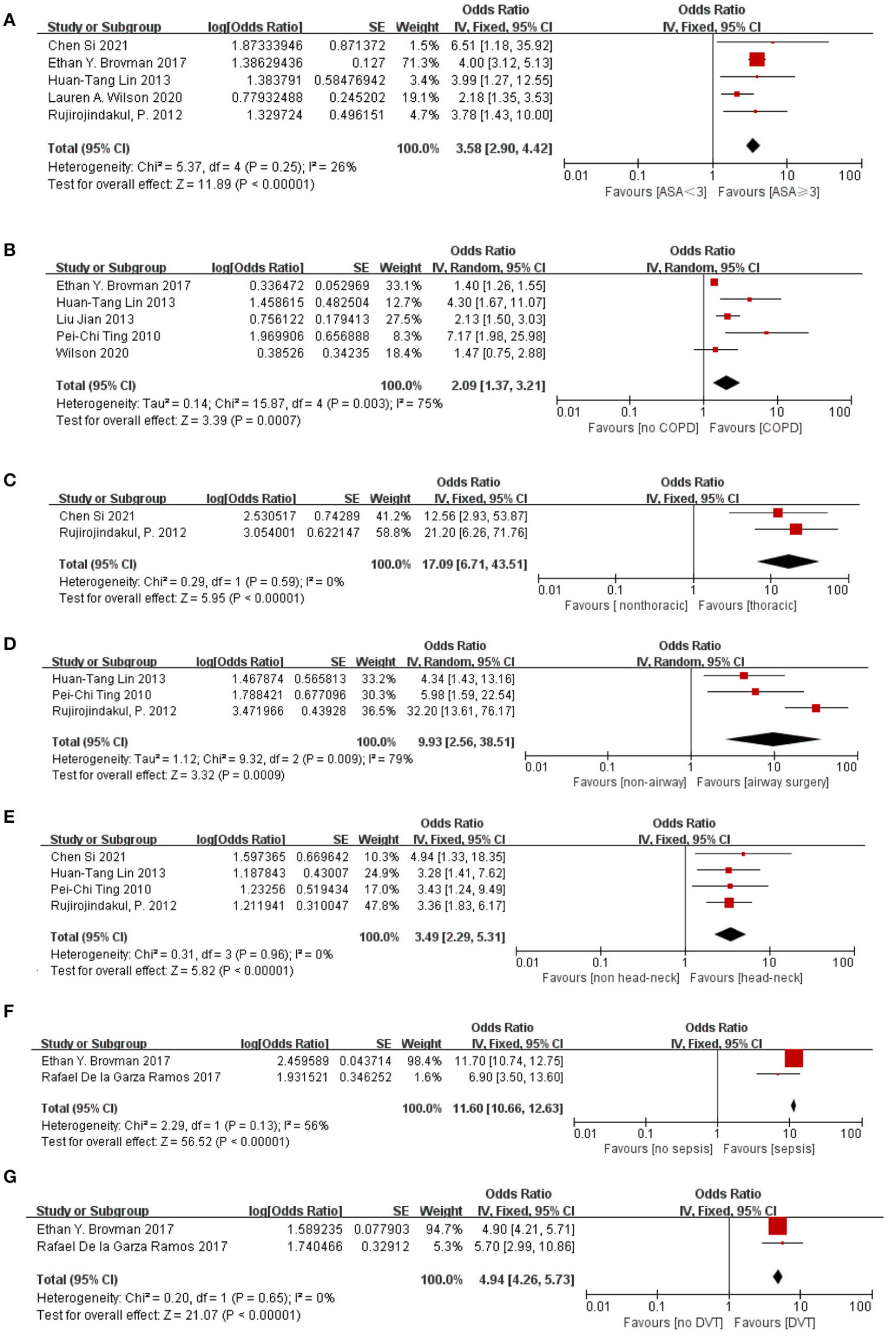
Forest plot of the association between POR after GA and **(A)** ASA≥3, **(B)** COPD, **(C)** thoracic surgery, **(D)** airway surgery, **(E)** head-and-neck surgery, **(F)** sepsis and **(G)** DVT. The individual block squares denote the OR, the area of the squares is proportional to the weight of each study. The horizontal line represents 95% of the CI. The diamond denotes the pooled estimate and its 95% confidence interval. The diamonds drawn on the right section indicate an increased risk of POR. OR, odds ratio; CI, confidence interval.

Table 4 shows the risk factors for POR after GA in our meta-analysis. All risk factors were binary variables. I^2^ statistics were used to evaluate the degree of statistical heterogeneity. The following 7 risk factors for POR were significantly different: ASA≥ (OR = 3.58, 95%CI 2.90–4.42), COPD (OR = 2.09, 95%CI 1.37–3.21), thoracic surgery (OR = 17.09, 95%CI 6.71–43.51), airway surgery (OR = 9.93, 95%CI 2.56–38.51), head-and-neck surgery (OR = 3.49, 95%CI 2.29–5.31), sepsis (OR = 3.50, 95%CI 2.21–5.55). DVT (OR = 4.94, 95%CI 4.26–5.73). Figure 2 shows the forest plot describing the relationship between the 7 risk factors and POR.

**Table 4:** Meta-analysis of risk factors of POR in patients after GA.

**Risk factors**	**Combination** **studies**	**Cases/controls**	**OR (95%CI)**	**Z**	**P**	**Heterogeneity of study design**			**Analysis model**	**Harbord's test**
						**χ^2^**	***P***	***I* ^2^**		
Male gender	4	7111/357395	1.05 (0.87, 1.25)	0.49	0.63	6.44	0.09	53	Random	0.031
Age>65	2	200/800	2.62 (0.36, 19.24)	0.95	0.34	8.17	0.004	88	Random	NA
ASA≥3	5	7266/358219	3.58 (2.90, 4.42)^a^	11.89	<0.001	5.37	0.25	26	Fixed	0.012
COPD	5	7346/358815	2.09 (1.37, 3.21)^a^	3.39	<0.001	15.87	0.003	75	Random	0.07
Rocuronium	2	169/612	1.38 (0.94, 2.03)	1.62	0.11	2.09	0.15	52	Random	NA
Emergency case	3	7139/310582	1.47 (0.87, 2.47)	1.45	0.15	27.21	<0.001	93	Random	0.298
Thoracic surgery	2	200/800	17.09 (6.71, 43.51)^a^	5.95	<0.001	0.29	0.59	0	Fixed	NA
Airway surgery	3	377/1295	9.93 (2.56, 38.51)^a^	3.32	<0.001	9.32	0.009	79	Random	0.403
Head-and-neck surgery	4	413/1439	3.49 (2.29, 5.31)^a^	5.82	<0.001	0.31	0.96	0	Fixed	0.063
Perioperative sepsis	2	7118/319256	10.05 (6.29, 16.06)^a^	9.64	<0.001	2.29	0.13	56	Random	NA
Perioperative DVT	2	7118/319256	4.94 (4.26, 5.73)^a^	21.07	<0.001	0.20	0.65	0	Fixed	NA

a *< 0.05 stands for significant*.

### PARP of Risk Factors

**Figure 3:**
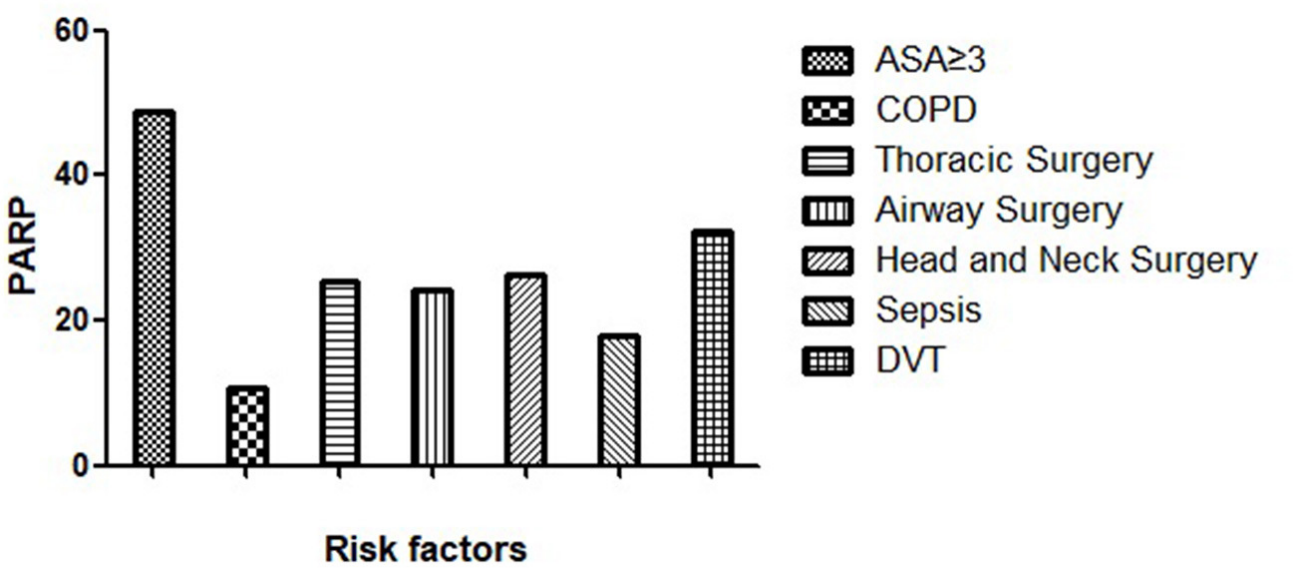
Bar chart of PARP of risk factors. Each column on the X-axis represents a risk factor, and the height of the Y-axis represents the size of the PARP.

The percentage of cases attributable to exposure factors in the population was estimated using PARP. This meta-analysis calculated the PARP of risk factors of binary variables as shown in Figure 3. Results were as follows: ASA ≥ 3 (PARP = 48.8%), airway surgery (PARP = 24.1%), head-and-neck surgery (PARP = 26.4%), thoracic surgery (PARP = 25.5%), COPD (PARP = 10.7%), sepsis (PARP = 17.9%), DVT (PARP = 3.2%). The results showed that ASA ≥ 3 was the most important risk factor for POR after GA.

### Sensitivity Analysis

**Figure 4:**
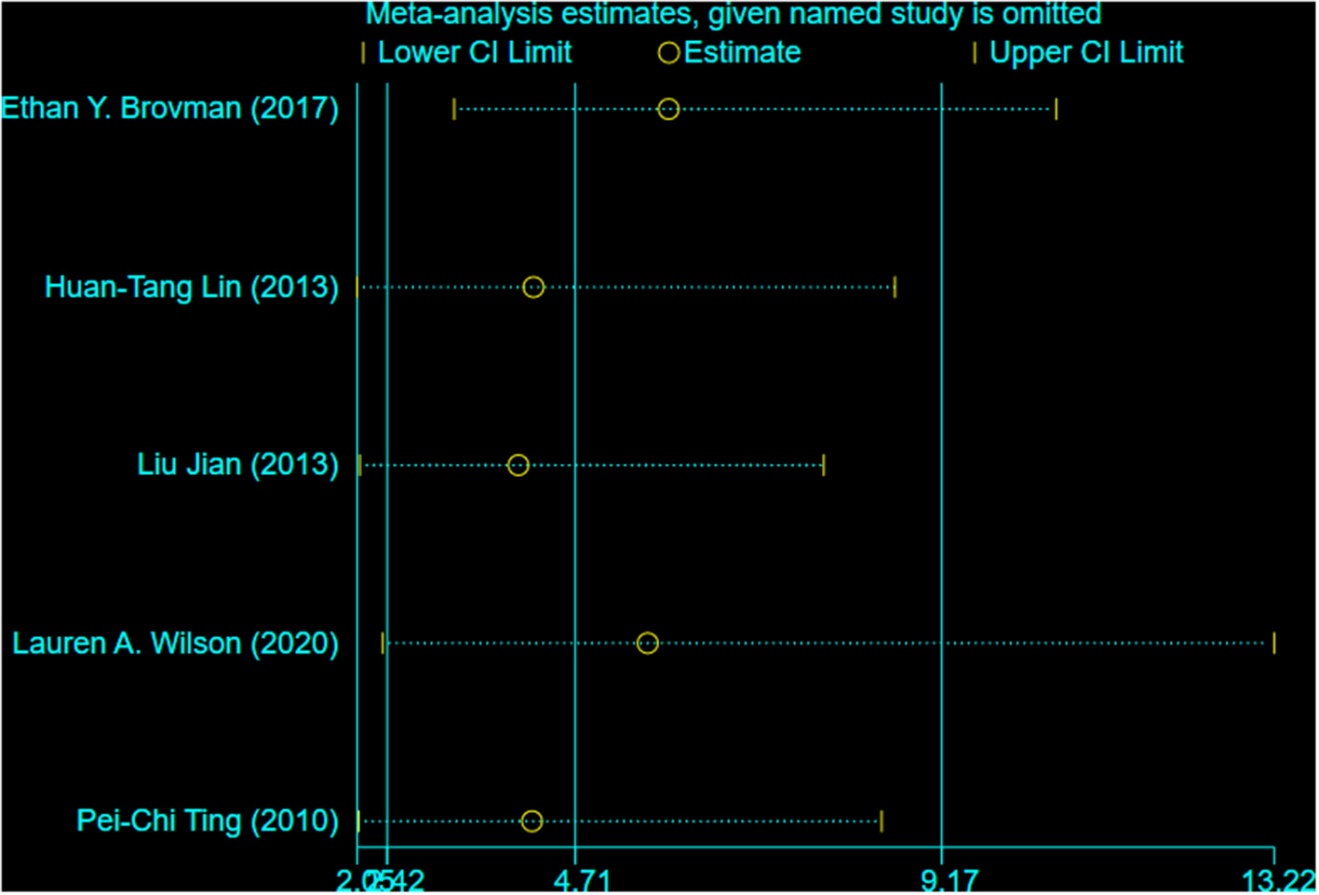
Sensitivity analysis diagram of COPD.

In the analysis of COPD, we carried out a sensitivity analysis by excluding each study one by one to explore whether a study significantly impacts the results or whether it has a significant contribution to heterogeneity (Figure 4). Overall, we found that the results were not affected by any study and that our meta-analysis was relatively robust. However, after excluding the study by Ethan et al. and Pei-Chi Ting et al. the heterogeneity decreased significantly, indicating that the two studies were the main source of heterogeneity.

### Publication Bias

There was little evidence of publication bias regarding airway surgery, as indicated by Harbord's test (*P* = 0.403). However, we detected publication bias in ASA ≥ 3, COPD and Head-and-Neck Surgery (*P* = 0.012, *P* = 0.07, *P* = 0.063).

## Discussion

### Main Findings

In recent years, the incidence of POR after GA has decreased with improvements in the medical field. However, the occurrence of POR remains an issue that needs to be tackled since it is closely related to the poor prognosis of patients. To better identify the patients at risk for POR, we performed a meta-analysis by including data from 10 studies to systematically analyze risk factors for POR in patients following GA. We only included studies deemed as high quality using the NOS scale to reinforce the credibility and robustness of our results. After carefully analyzing and evaluating the 10 studies, we identified 7 risk factors related to POR for patients following GA, including ASA ≥ 3, COPD, thoracic surgery, airway surgery, head-and-neck surgery, sepsis, and DVT.

### Explanation of Results

Our evaluation showed that the risk of POR was 3.58 times higher in patients with ASA ≥ 3 before general anesthesia than in patients with ASA < 3. ASA classification is a commonly used index for pre-anesthesia risk assessment of patients formulated by the American Medical Association, and the higher the rating, the worse the health status of patients (21). Patients with ASA ≥ 3 classification often have more serious systemic diseases and limited physical activities. The preoperative status of the patient is a key determinant of postoperative recovery. Therefore, the physical condition of a patient plays an important role in the recovery after general anesthesia.

Consistent with the clinical setting, our meta-analysis demonstrated that patients with COPD had a 2.09-fold higher risk of POR than patients without COPD. The pathogenesis of POR in COPD patients may be attributed to the abnormal function of the small airways, which leads to increased airway resistance and aggravates respiratory muscle weakness during anesthesia recovery (16). Most preoperative COPD patients had difficulties in postoperative extubation; therefore, clinicians should strictly grasp the indications of tracheal extubation in elderly COPD patients, and ventilator-assisted ventilation should be used when necessary (22). Hence, greater consideration should be given to COPD patients during preoperative evaluation, and more caution is warranted during perioperative management.

The surgical site was also reported to be closely related to POR occurrence in patients after GA (18, 23). Similar to the results of previous studies (20, 24), our study suggested that thoracic surgery, airway surgery, and head-and-neck surgery were risk factors for POR. Thoracic surgery, especially pneumonectomy, decreases lung volume and gas exchange area. Besides, postoperative indwelling thoracic drainage tubes and poor postoperative analgesia often increase patients' oxygen consumption. Over-tightening of chest straps could lead to a decrease in sputum excretion capacity and a reduction in effective pulmonary ventilation, even leading to hypoxemia or hypercapnia (25). In addition, a study showed that up to 11% of patients with POR after thoracic surgery developed phrenic nerve injury (1). Patients undergoing airway surgery are generally suffering from respiratory diseases, such as obstructive sleep apnea syndrome. Besides, these patients usually have an abnormal airway morphology, and surgical compression during surgery makes them prone to airway edema, so the probability of POR in such patients is greatly increased (26). A study has shown that the high risk of POR in head-and-neck surgeries may be related to the proximity of the operating field to the respiratory tract or the “shared airway” (27). Therefore, for postoperative patients undergoing thoracic surgery, airway surgery, and head-and-neck surgery, the anesthesiologist should conduct adequate communication and handovers with the surgeon and the nurse in the anesthesia recovery room to ensure that the above patients undergo a comprehensive pre-extubation evaluation.

Our study also identified that POR risk in patients with perioperative DVT was 4.94 times higher than that in patients without DVT. It might be those patients were reintubated due to respiratory failure secondary to Pulmonary emboli. A previous study showed that endotracheal intubation was a risk factor for deep venous thrombosis due to the reduced mobility of those patients (28). Therefore, it is reasonable to believe that POR and DVT are mutually causal.

Moreover, our results showed that POR risk in patients with perioperative sepsis was 10.05 times higher than that in patients without sepsis. Sepsis is one of the important predictors of perioperative respiratory failure and death, suggesting the importance of preventing infections in preoperative patients (29). Once sepsis occurs, we should actively treat it and choose appropriate general anesthetic drugs for anti-inflammation.

Interestingly, our findings found no association between age >65 and POR after GA. However, we still need to be vigilant about the perioperative care of surgical patients ≥65 years old since it was shown to be an important risk factor for failed extubation in ICU patients (30). Elderly patients have a weaker tolerance to intraoperative stimulation and metabolize anesthetic drugs slower due to the degeneration of various functions of the body. The longer recovery time needed after general anesthesia often leads to delayed extubation; hence more vigilance is warranted. Similarly, the results of this meta-analysis showed that rocuronium was not a risk factor for POR, which may be due to the inclusion of only two small sample size studies. In a previous randomized controlled study, rocuronium had a longer action time and a longer metabolic time than cis-atracurium and was more likely to induce POR (31).

Herein, we used PARP to estimate the percentage of POR related to risk factors in patients after general anesthesia. This study showed that PARP was the highest in the ASA ≥ 3 group. Therefore, we conclude that it is an important risk factor for POR after general anesthesia. The PARP for sepsis was 17.9%, and the PARP for DVT was only 3.2%. Nevertheless, they are two preventable factors, and measures should be taken to prevent sepsis and DVT in patients after surgery.

### Implication for Clinical Practice

Many studies have utilized early extubation after surgery to shorten mechanical ventilation and ICU stay times (32, 33). Nonetheless, early extubation can lead to POR, further prolonging ICU stay time and increasing hospital mortality rates. Therefore, risk stratification is recommended for surgical patients to maximize the success rate of early extubation. The ASA classification is an important tool to reflect patients' baseline physical condition status and severity that should never be disregarded. In addition, anesthesiologists, surgeons, and nurses must consider these risk factors during the perioperative management of such patients to optimize general status, pulmonary function and supplement analgesia if needed. Furthermore, anesthesiologists should consider possibly delaying extubation or transferring such patients to the ICU postoperatively. Decreasing the incidence of PORs will ultimately decrease medical cost, morbidity, and mortality in the perioperative setting.

### Implication for Future Studies

Among the included studies, no study used respiratory post-op complications score as a predictor of POR, and the predictive value of the score for POR can be further explored in the future. Moreover, It is necessary to use the identified risk factors to establish a prediction model to improve the risk stratification of POR. A well-designed large sample cohort study and multivariate regression analysis are warranted in the future. Further interventional research should be carried out according to the prediction model instead.

### Limitations of the Study

Our meta-analysis has some limitations. First of all, only studies published in English were included. Secondly, a study (Ethan et al.) dominated the results as it included 80% of studied cases,which have played a big part in our conclusions. Finally, the summarized results were based on 10 or fewer studies since relatively few studies investigated the risk factors associated with POR after GA. Therefore, it is necessary to conduct more carefully designed studies on the potential risk factors of POR in patients after GA.

## Conclusions

In conclusion, this meta-analysis identified some risk factors for POR in patients after GA and provided a reference for the prevention of POR. However, more strictly designed prospective cohort studies are needed to substantiate our findings and further identify effective measures to control POR.

## Data Availability Statement

The original contributions presented in the study are included in the article/supplementary material, further inquiries can be directed to the corresponding authors.

## Author Contributions

ZX made substantial contributions to conception and design of the study. JL and SW conducted the search. YD and LY screened articles. JL and LT conducted the data extraction and study quality assessment. ZX analyzed the data and wrote the manuscript. ZY and LT revised the manuscript. All the authors approved the final version of the manuscript.

## Funding

This research was supported by Science and Technology Department of Jiangxi Province, Grant/Award Numbers: 20192BBGL70016 and 20181BBG78028. The funder were not involved in study design, data collection, analysis and interpretation, report writing, and publication. All researchers are completely independent of the funders.

## Conflict of Interest

The authors declare that the research was conducted in the absence of any commercial or financial relationships that could be construed as a potential conflict of interest.

## Publisher's Note

All claims expressed in this article are solely those of the authors and do not necessarily represent those of their affiliated organizations, or those of the publisher, the editors and the reviewers. Any product that may be evaluated in this article, or claim that may be made by its manufacturer, is not guaranteed or endorsed by the publisher.

## Abbreviations

ASA: American Society of Anesthesiologists
CI: Confidence interval
COPD: Chronic obstructive pulmonary disease
CPR: Cardiopulmonary resuscitation
DVT: Deep venous thrombosis
ICU: Intensive care unit
LOS: Length of stay
OR: Odds ratio
PARP: Population attribution risk ratio.
