# Supplementary material for: Risk Factors for Post-operative Planned Reintubation in Patients After General Anesthesia: A Systematic Review and Meta-Analysis
Source: Front Med (Lausanne). 2022 Mar 9;9:839070. doi: 10.3389/fmed.2022.839070 (PMC8959864; doi:10.3389/fmed.2022.839070)
Supplement: Supplementary file 1 [file Data_Sheet_1.docx]

**Pubmed**

**#1 (reintubation[Title/Abstract]) OR (re-intubation[Title/Abstract]) 2735**

**#2 (((factors[Title/Abstract]) OR (risk factor[Title/Abstract])) OR (influence factor[Title/Abstract])) OR (relevant factor[Title/Abstract]) 2470943**

**#3 ((Anesthesia[Title/Abstract]) OR (surgery[Title/Abstract])) OR (operative[Title/Abstract]) 1646586**

**#1 and #2 and #3 367**

**Web of science**

**#1 TS=(reintubation OR re-intubation) 2291**

**#2 TS=(factors OR risk factor OR influence factor OR relevant factor) 5607699**

**#3 TS=(Anesthesia OR surgery OR operative) 1412502**

**#1 and #2 and #3 442**

**Embase**

**#1 reintubation:ab,ti OR 're intubation':ab,ti 4799**

**#2 factors:ab,ti OR 'risk factor':ab,ti OR 'influence factor':ab,ti OR 'relevant factor':ab,ti 3224944**

**#3 anesthesia:ab,ti OR surgery:ab,ti OR operative:ab,ti 2188798**

**#1 and #2 and #3 651**

**Cochrane**

**
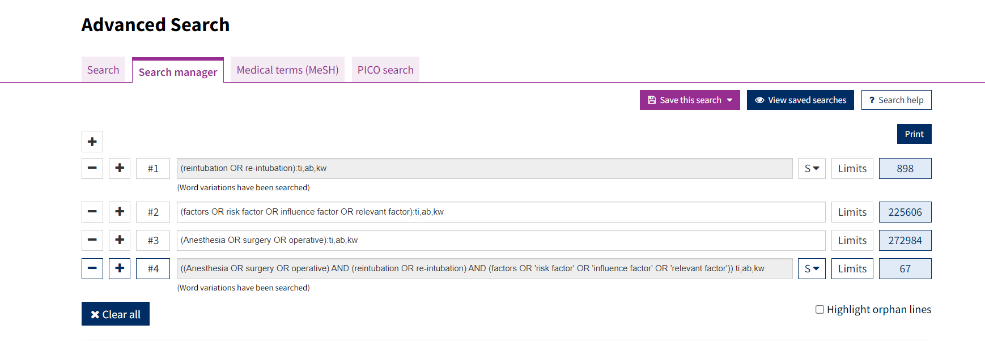
**

**#1 898**

**#2 225606**

**#3 272984**

**#1 AND #2 AND #3 67**

**Total:367+442+651+67=1527**

**registration number
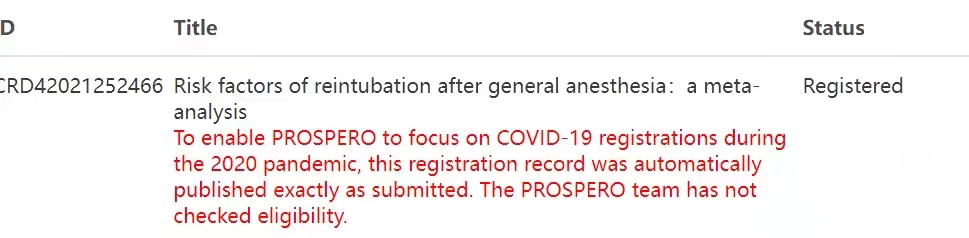
**

**Quality assessment**

| **Study** | **Selection** | **Comparability** | **Outcome** | **Total scores** |
| --- | --- | --- | --- | --- |
| Chen Si(1) | **☆☆☆☆** | **☆☆** | **☆☆☆** | 7 |
| Ethan Y. Brovman(3) | **☆☆☆☆** | **☆☆** | **☆☆☆** | 6 |
| Huan-Tang Lin(17) | **☆☆☆☆** | **☆☆** | **☆☆☆** | 7 |
| Karla M. Greco(15) | **☆☆☆☆** | **☆☆** | **☆☆☆** | 8 |
| Lauren A. Wilson(19) | **☆☆☆☆** | **☆☆** | **☆☆☆** | 7 |
| Liu Jian(16) | **☆☆☆☆** | **☆☆** | **☆☆☆** | 7 |
| Pei-Chi Ting（20） | **☆☆☆☆** | **☆☆** | **☆☆☆** | 6 |
| Rafael De la Garza(4) | **☆☆☆☆** | **☆☆** | **☆☆☆** | 8 |
| Rafael De la Garza(14) | **☆☆☆☆** | **☆☆** | **☆☆☆** | 7 |
| Rujirojindakul, P.(18) | **☆☆☆☆** | **☆☆** | **☆☆☆** | 8 |

**Note: A study can be awarded a maximum of one star for each numbered item within the Selection and Exposure categories. A maximum of two stars can be given for Comparability.** **A red star☆ indicates that the item is not eligible.**
